# Real-Time Ultrasound-Guided Transurethral Incision for Posterior Urethral Valves

**DOI:** 10.3390/children12101365

**Published:** 2025-10-09

**Authors:** Yudai Goto, Kouji Masumoto, Takato Sasaki, Yasuhisa Urita, Kazuki Shirane, Katsuhiko Ueoka

**Affiliations:** 1 Department of Pediatric Surgery, Faculty of Medicine, University of Tsukuba, Tsukuba 305-8577, Japan; kmasu@md.tsukuba.ac.jp (K.M.); ta-sasaki@md.tsukuba.ac.jp (T.S.); y-urita@md.tsukuba.ac.jp (Y.U.); shirane.kazuki.nh@ms.hosp.tsukuba.ac.jp (K.S.); katsueoka@gmail.com (K.U.); 2 Department of Pediatric Surgery, Tsukuba Memorial Hospital, Tsukuba 300-2622, Japan

**Keywords:** Posterior urethral valves, transurethral incision, real-time ultrasound-guided transurethral incision, E/C ratio

## Abstract

**Highlights:**

**What are the main findings?**

- Real-time ultrasound-guided transurethral incision (RUG-TUI) allowed direct intraoperative visualization of the urethral longitudinal axis and the anatomical relationship between the stenosis and external urethral sphincter.
- RUG-TUI significantly shortened postoperative hematuria duration and enables quantitative evaluation of incision effectiveness using the E/C ratio.

**What is the implication of the main finding?**

- Integrating real-time ultrasound guidance into PUV surgery may help prevent sphincter injury and reduce unnecessary deep incisions.
- Intraoperative E/C ratio measurement offers an objective method to assess treatment adequacy, potentially decreasing the need for reoperation.

**Abstract:**

**Background/Objectives:** Transurethral incision (TUI) is a common procedure for posterior urethral valves (PUV). However, no standardized method has been established to assess its efficacy intraoperatively. In this study, we aimed to develop and evaluate a real-time ultrasound-guided TUI (RUG-TUI) technique. **Methods**: A single-center, retrospective feasibility study with a cohort design was conducted using historical controls to compare RUG-TUI with standard TUI in children with PUV. Data from patients who underwent RUG-TUI for PUV between April 2021 and July 2022 or TUI without real-time ultrasound guidance between August 2020 and March 2021 (control group) were retrospectively reviewed. A transperineal linear probe provided longitudinal imaging. The diameters of the constricted (C) and expanded (E) portions of the urethra before and after the procedure were measured, and the E/C ratio was calculated. The primary outcome was the duration of postoperative gross hematuria, and the secondary outcomes included changes in the urethral diameter ratio (E/C ratio), intraoperative complications, and residual obstruction on VCUG. **Results**: The mean duration of post-procedure macroscopic hematuria was significantly shorter in the RUG-TUI group than in the control group (*p* = 0.049). No massive intraoperative bleeding or sphincter damage occurred. In the RUG-TUI group, the mean diameters of the constricted segment before and after the procedure were 3.0 (±1.0) and 5.7 (±1.2) mm, respectively, while the pre- and postoperative E/C ratios were 1.8 (±0.5) and 0.9 (±0.1), respectively (*p* < 0.0001). **Conclusions**: RUG-TUI for PUV enabled visualization of the longitudinal axis of the urethra, allowing assessment of the anatomical relationship between the stenosis and external urethral sphincter. In this retrospective feasibility cohort, RUG-TUI was associated with a shorter duration of postoperative gross hematuria. These exploratory findings suggest that RUG-TUI may support intraoperative evaluation of procedural adequacy.

## 1. Introduction

Posterior urethral valves (PUV) cause urethral obstruction in pediatric male patients, affecting approximately 1/5000–8000 children [1], and the severity of PUV varies. Neonates with severe PUV have a prenatal history of acute or recurrent pyelonephritis, along with bilateral hydronephrosis or significant vesicoureteral reflux, requiring surgical intervention to prevent deterioration of renal function [2]. Conversely, patients with mild PUV often experience delayed diagnosis and commonly present with daytime urinary incontinence and nocturnal enuresis as their chief complaints [3]. PUV have also been reported to be more common with hypospadias [4]. Therefore, bilateral hydronephrosis, recurrent urinary tract infections, specific forms of hypospadias, and urinary incontinence are common indications for a voiding cystourethrogram (VCUG) to diagnose PUV. Although no clear criteria exist for determining the degree of PUV that necessitates surgical intervention, surgical treatment is known to alleviate clinical symptoms even in patients with urinary incontinence and nocturnal enuresis [5].

Imaging characteristics of PUV include bladder wall thickening, dilation of the posterior urethra, and non-distention of the anterior urethra [1]. This is based on the concept that the posterior urethra can stretch with increased voiding pressure owing to its unique histological and anatomical configuration, unlike the anterior urethral segment [6].

Except in severe or neonatal cases, where a temporary vesicostomy or ureterostomy may be performed, the current gold-standard treatment for PUV is endoscopic transurethral incision (TUI). Although the outcomes of TUI have improved with advancements in surgical equipment and medical practice, the incidence of residual valves or urethral strictures necessitating re-resection is between 7% and 25% [7].

A consensus exists regarding postoperative evaluation using VCUG, kidney function monitoring, hydronephrosis assessment, vesicoureteral reflux, urinary stream, and bladder emptying to evaluate the efficacy of PUV treatment [8]. However, these parameters often change gradually over several months, and both cystoscopy and VCUG have disadvantages: cystoscopy is the diagnostic gold standard for direct visualization but is invasive in pediatric settings [8], while VCUG exposes the patient to radiation [9]. These limitations highlight the need for a method to assess the adequacy of valve incision intraoperatively.

We developed a novel surgical technique, real-time ultrasound-guided TUI (RUG-TUI), to enable intraoperative ultrasound evaluation of treatment efficacy. In this preliminary retrospective study, we aimed to report this technique and the results of utilizing RUG-TUI, as well as to evaluate its potential as a method for assessing the efficacy of PUV treatment.

## 2. Materials and Methods

This was a single-center, retrospective feasibility study with a cohort design, using a non-concurrent historical control. Outcomes of consecutive children undergoing RUG-TUI were compared with those of a previous cohort treated with standard TUI under the same inclusion/exclusion criteria.

### 2.1. Study Population

Patients diagnosed with PUV who underwent VCUG and RUG-TUI between April 2021 and July 2022 were retrospectively reviewed. Patients who did not undergo VCUG after the procedure or whose urethral diameters could not be measured using preoperative and postoperative ultrasound imaging were excluded. For the control group, patients who underwent TUI without real-time ultrasound guidance between August 2020 and March 2021 were retrospectively reviewed. In both groups, only patients who had ≥24 months of postoperative follow-up were included. For the distal outcome, events were ascertained within a fixed 24-month window, and patients with longer follow-up were administratively censored at 24 months.

Parameters obtained from medical records, preoperative VCUG, postoperative VCUG at approximately 6 months, surgical videos, and ultrasound images were analyzed. These included conditions associated with PUV, frequency of difficult intraoperative bleeding, frequency of external sphincter injury, procedure time, duration of postoperative gross hematuria, and improvement of PUV on the 6-month postoperative VCUG. Postoperative VCUG images were independently evaluated by at least two board-certified pediatric surgeons. Improvement of posterior urethral valves was assessed comprehensively based on the presence or absence of posterior urethral dilation and the overall voiding pattern.

### 2.2. Surgical Procedures

**Figure 1:**
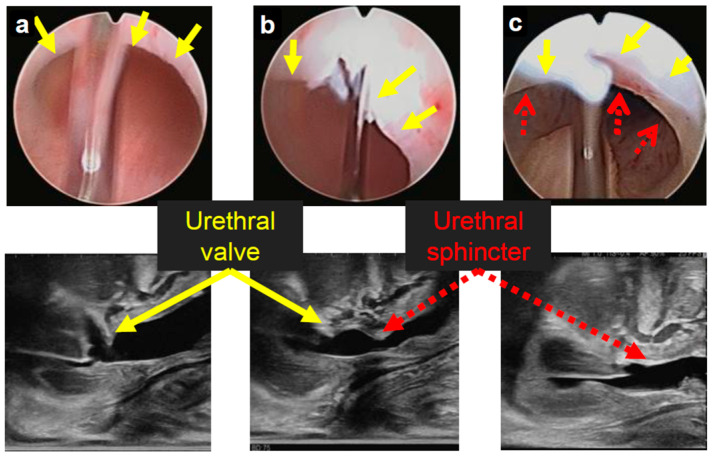
Comparison of urethral ultrasound and urethroscopic findings in the same patient. (**a**) PUV is observed on the ventral side (solid-line arrow). (**b**) Initial incision of PUV. Although the urethral sphincter is not visible in the urethroscopic image, it is shown on ultrasound image in close proximity to the PUV (dashed-line arrow). (**c**) Both the urethroscopic and ultrasound images confirm the presence of the urethral sphincter. PUV: posterior urethral valves.

**Figure 2:**
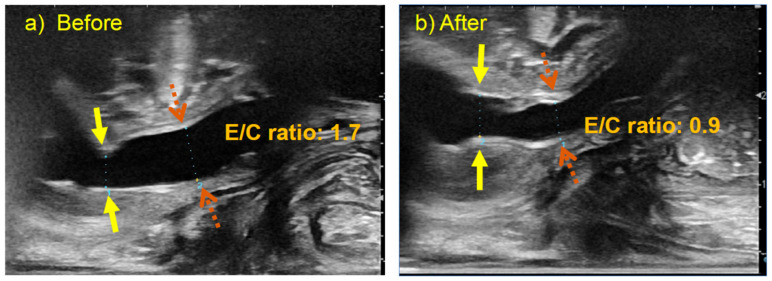
Comparison of urethral ultrasound images before and after urethral incision in a 2-year-old with PUV. Yellow solid arrows indicate the constricted (C) portion, and brown dashed arrows indicate the expanded (E) portion of the urethra. (**a**) Ultrasound finding before the urethral incision. The posterior urethra is dilated because of PUV. Urethral diameter at the (C) and (E) were 3.3 mm, 5.7 mm, E/C ratio: 1.7. (**b**) Ultrasound finding after the urethral incision. The narrow portion of the urethra due to PUV is relieved. Urethral diameter at the (C) and (E) were 4.7 mm, 4.1 mm, E/C ratio: 0.9. PUV: posterior urethral valves, E/C ratio: ratio of the diameters of the constricted (C) and expanded (E) portions of the urethra.

RUG-TUI was performed on patients in the lithotomy position under general anesthesia with the appropriate administration of muscle relaxants. Initially, an 8.5-French resectoscope was inserted through the urethra to observe the urethra and bladder, after which normal saline was injected into the bladder. Following removal of the cystoscope, urethral diameters were measured in the mid-sagittal view using a transperineal linear probe with a frequency of 8–14 MHz (ARIETTA 70, Hitachi Aloka Medical, Ltd., Tokyo, Japan) while applying suprapubic pressure. The measurement sites were defined as the expanded posterior urethral segment (E) and the constricted segment (C) caused by the PUV before TUI. Subsequently, a sickle-shaped cold knife was attached to the resectoscope to perform a urethrotomy. At our institution, a single incision at the 12 o’clock position is performed as the standard approach. An assistant positioned a linear probe dorsally and applied it transperineally to avoid interfering with the surgeon. The ultrasonogram was inverted to facilitate comparison with the cystoscopy findings (Figure 1), and the two images were displayed in parallel on a monitor. The surgeon focused on performing the urethrotomy, while the assistant and ultrasound operator communicated the findings orally. At the end of the procedure, saline was injected into the bladder through the resectoscope. The resectoscope was then removed, and the urethral diameters of the C and E segments were measured using ultrasonography at the same locations as before the incision, while suprapubic pressure was applied to induce voiding. In addition, the ratio of the diameter of the E segment to that of the C segment of the urethra (E/C ratio) was calculated (Figure 2).

Following the incision, a catheter was placed in the bladder for bladder irrigation. The convex ultrasound probe was used to visualize the bladder via the transabdominal approach and confirm the clearance of blood clots. The catheter was removed on postoperative day 7, and all patients were discharged on postoperative day 8. All procedures in both the RUG-TUI and control groups were performed by board-certified pediatric surgeons.

This retrospective feasibility analysis was based on the hypothesis that RUG-TUI enables adequate valve incision without excessive depth, thereby reducing urethral damage and shortening the duration of gross hematuria compared to TUI without ultrasound guidance.

The primary outcome was the duration of postoperative gross hematuria, defined as the number of days from the end of surgery to the last calendar day on which visible hematuria was documented in nursing logs or clinical notes. This outcome was selected as a proxy for intraoperative urethral injury. Pre-specified secondary outcomes included: (i) the presence of residual PUV or persistent obstruction on postoperative VCUG; (ii) the need for additional procedures for residual obstruction during follow-up; (iii) intraoperative bleeding and other procedure-related complications, including suspected injury to the external sphincter; (iv) the change in E/C ratio from pre- to post-incision in the RUG-TUI group, reported as a feasibility and process indicator.

Treatment efficacy was defined using both clinical and imaging parameters. The primary efficacy endpoint was the duration of gross hematuria, and the key secondary endpoint was the absence of residual PUV on postoperative VCUG. Since this study was designed as a retrospective feasibility cohort, between-group comparisons were exploratory and not powered for confirmatory analysis.

Statistical analysis was performed by Mann–Whitney U and Wilcoxon matched-pairs signed rank tests using GraphPad Prism, version 9.5.1 for Windows (GraphPad Software, San Diego, CA, USA, www.graphpad.com). Statistical significance was set at *p* > 0.05.

This study was approved by the Institutional Review Board of the University of Tsukuba Hospital, which serves as the primary governing body for medical research (approval number: R05-068). Additionally, patients were allowed to withdraw from the study through our website if they wanted. Given the noninvasive and observational nature of our study, the ethics committee granted permission to waive the requirement for written informed consent from each patient.

## 3. Results

During the study period, 32 patients underwent RUG-TUI. Twelve who did not undergo postoperative VCUG and four whose preoperative and postoperative urethral diameters could not be measured were excluded, leaving a final sample size of 16. The control group comprised 21 patients who underwent urethrotomy without real-time ultrasound guidance. Five patients who did not undergo postoperative VCUG were excluded, resulting in a final sample size of 16. The demographic data are shown in Table 1.

The mean duration of postoperative gross hematuria was significantly shorter in the RUG-TUI group than in the control group (Table 1). There were no significant differences in age at the time of the procedure and median procedure time between the RUG-TUI and control groups (Table 1). No cases of difficult-to-control bleeding or obvious sphincteric damage during the procedure were observed. The improvement rate on postoperative VCUG was 100% in both groups, and no patients required a second incision. No cases of apparent urinary incontinence were observed in either group during the postoperative follow-up period.

**Figure 3:**
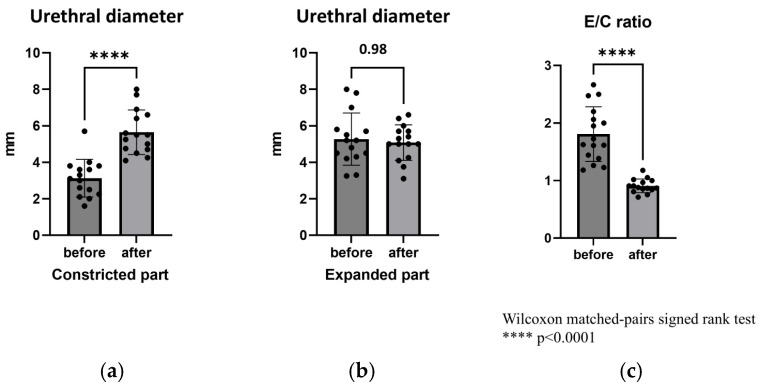
Diameters and E/C ratio before and after RUG-TUI. (**a**) The median urethral diameters (before and after RUG-TUI were 3.1 mm (range, 1.6–5.7 mm) and 5.5 mm (range, 4.1–8.0 mm). (**b**) The median urethral diameters (E) before and after RUG-TUI were 5.0 mm (range, 3.3–8.0 mm) and 5.0 mm (range, 3.1–6.6 mm). (**c**) The E/C ratios before and after RUG-TUI were 1.7 (range, 1.2–2.7) and 0.9 (range, 0.7–1.2). E/C ratio: ratio of the diameter of the expanded (E) portion to that of the constricted (C) portion of the urethra, RUG-TUI: real-time ultrasound-guided transurethral incision.

Furthermore, the diameters of the C and E segments of the urethra before and after RUG-TUI were compared. The median preoperative diameters of the E and C segments of the urethra were 5.0 mm (range, 3.3–8.0 mm) and 3.1 mm (range, 1.6–5.7 mm), respectively, with a median E/C ratio of 1.7 (range, 1.2–2.7). The median postoperative diameters of the E and C segments of the urethra were 5.0 mm (range, 3.1–6.6 mm) and 5.5 mm (range, 4.1–8.0 mm), respectively, with a median E/C ratio of 0.9 (range, 0.7–1.2). The diameter of the C segments and the E/C ratio revealed a significant difference between the preoperative and postoperative measurements (*p* < 0.0001) (Figure 3).

## 4. Discussion

To the best of our knowledge, this single-center, retrospective feasibility study with a cohort design using historical controls is among the first to describe RUG-TUI for PUV in children. This procedure combines ultrasound and urethroscopy, enabling the visualization of the longitudinal axis of the urethra and facilitating easy assessment of the anatomical relation of the stenosis and external urethral sphincter. Furthermore, our results suggest that RUG-TUI may reduce the duration of postoperative hematuria and facilitate an accurate evaluation of treatment effectiveness during the procedure on a real-time basis.

In ultrasonography, the depiction of PUV is the most effectively achieved in the longitudinal plane, where they appear as mobile and linear hyperechoic structures in the posterior urethra [10]. Reports using ultrasonography with contrast agents have described typical findings in children with PUV, such as a dilated posterior urethra and a thin thread extending to the anterior urethra, indicative of impaired flow into the anterior urethra, thereby enabling the diagnosis of PUV [9]. However, the sensitivity of ultrasonography has depended on the experience of operators [11]. In our study, intraoperative ultrasonography was conducted by board-certified pediatric surgeons who regularly perform the procedure in their clinical practice. Although no contrast agent was used in our study, as illustrated in Figure 1, the cystoscope appeared as a distinct hyperechoic structure within the urethra, facilitating the identification of the urethra.

One complication of PUV treatment is damage to the external urethral sphincter, which can cause urinary incontinence after the procedure [12]. In contrast, according to Duckett and Snow, 20–30% of patients require a second treatment for undercutting of PUV. They stated that undercutting, rather than overcutting, is essential to reduce the risk of urethral stricture and urinary incontinence [13]. As shown in Figure 1, the narrowed part and the sphincter are often in close proximity. Given the elevated risk of sphincter injury during the early stages of incision, integrating long-axis ultrasound imaging may be instrumental in preventing these complications.

Furthermore, RUG-TUI permits a more detailed macroscopic evaluation and enables estimation of the effectiveness of the incision. Good et al. reported the usefulness of transabdominal ultrasound during voiding for the diagnosis of PUV, with sensitivity, specificity, and positive predictive values of 100%, 89%, and 88%, respectively, and a posterior urethral diameter of 6 mm or greater [10]. However, the challenge encountered when applying this criterion during urination under general anesthesia with suprapubic pressure is that the urethral diameter varies depending on the pressure exerted on the urethra. Only three of the 16 patients (18.8%) met this criterion despite a preoperative VCUG diagnosis of PUV. Therefore, we evaluated the urethral diameter and calculated the ratio of the stenotic to the dilated segment. Although a widespread consensus does not exist, reports since 2006 have suggested that the posterior-to-anterior urethral ratio (PAR) measured using VCUG is useful for evaluating postoperative outcomes [6,8,11,14,15]. These studies have demonstrated that urethral measurements can vary depending on the voiding stage, voiding volume, patient position, and measurement point. Although PAR values ranging from 2.2 to 3.5 have been reported as the cutoff for the success of the procedure [14], direct comparison of these values is challenging owing to variations in measurement conditions and imaging modalities. However, considering the subjective nature of assessing valve ablation efficacy based on a satisfactory urine stream in post-valve ablation [15], measuring the E/C ratio using ultrasound during the procedure, and assessing the real-time effectiveness of urethral incision would be beneficial.

RUG-TUI may shorten the duration of postoperative hematuria, potentially by reducing inappropriately deep incisions. The incidence of bleeding as a complication of external urethrotomy for PUV is unknown; however, RUG-TUI enables urethrotomy performance while potentially preventing unnecessary injuries and bleeding. In addition, ultrasound is considered effective for bladder irrigation following urethrotomy because it enables the visualization of blood clots, which may help reduce postoperative complications such as urinary catheter blockage.

Our study has some limitations that should be considered. First, this was a single-center, retrospective observational study. Due to the small sample size and use of historical controls, potential confounding factors such as patient background characteristics and differences between surgical operators cannot be ruled out. Second, postoperative evaluation was performed using only VCUG. Neither urodynamics nor uroflowmetry was used to assess urinary function, which may have provided more comprehensive information on the treatment efficacy. Third, the long-term outcomes of this procedure, such as kidney function and the frequency of urinary tract infections, remain unclear due to the limited follow-up period and require further investigation. Fourth, urethral ultrasonography is an operator-dependent modality, and variability related to probe pressure or technique may affect the accuracy of measurements. Although the E/C ratio was used to minimize these effects, intra- and interobserver variability was not assessed in this study. As this was an exploratory feasibility study, further research is needed to confirm the reproducibility and objectivity of this method.

## 5. Conclusions

In this retrospective feasibility study with a cohort design using historical controls, RUG-TUI enabled visualization of the longitudinal axis of the urethra, allowing intraoperative assessment of the anatomical relation between the stenosis and the urethral sphincter. RUG-TUI was associated with a shorter duration of postoperative gross hematuria. The ability to assess incision effectiveness intraoperatively may help reduce the need for reoperation. However, further studies are required to validate these findings.

## Figures and Tables

**Table 1:** Comparative analysis of patient characteristics between the RUG-TUI and TUI groups.

		RUG-TUI	TUI	*p* Value
Total number		16	16	
Co-morbidity				
	Vesicoureteral reflux (VUR)	7	11	
	Urinary tract infection (without VUR)	2	2	
	Hypospadias	4	0	
	Urinary incontinence	3	2	
	others (*)	0	1	
Age during procedure (month)				
	Median	24	36	0.180
	Range	6–112	6–137	
Procedure duration (min)				
	Median	42	50	0.513
	Range	24–49	22–63	
Duration of hematuria (days)				
	Median	1.5	3	0.048
	Range	1–6	1–6	

(*) VUR and urinary incontinence. RUG-TUI, real-time ultrasound-guided transurethral incision; TUI, transurethral incision.

## Data Availability

The datasets generated during and/or analyzed during the current study are available from the corresponding author upon reasonable request.

## Abbreviations

The following abbreviations are used in this manuscript:

PUV	Posterior urethral valve
VCUG	Voiding cystourethrogram
TUI	Transurethral incision
RUG-TUI	Real-time ultrasound-guided transurethral incision
PAR	Posterior-to-anterior urethral ratio
